# Association Between Peripheral Inflammation and DATSCAN Data of the Striatal Nuclei in Different Motor Subtypes of Parkinson Disease

**DOI:** 10.3389/fneur.2018.00234

**Published:** 2018-04-16

**Authors:** Hossein Sanjari Moghaddam, Farzaneh Ghazi Sherbaf, Mahtab Mojtahed Zadeh, Amir Ashraf-Ganjouei, Mohammad Hadi Aarabi

**Affiliations:** Faculty of Medicine, Tehran University of Medical Sciences, Tehran, Iran

**Keywords:** Parkinson disease, DATScan data, peripheral inflammation, motor subtypes, postural instability and gait difficulty, tremor-dominant, neutrophil to lymphocyte ratio

## Abstract

The interplay between peripheral and central inflammation has a significant role in dopaminergic neural death in nigrostriatal pathway, although no direct assessment of inflammation has been performed in relation to dopaminergic neuronal loss in striatal nuclei. In this study, the correlation of neutrophil to lymphocyte ratio (NLR) as a marker of peripheral inflammation to striatal binding ratios (SBRs) of DAT SPECT images in bilateral caudate and putamen nuclei was calculated in 388 drug-naïve early PD patients [288 tremor dominant (TD), 73 postural instability and gait difficulty (PIGD), and 27 indeterminate] and 148 controls. NLR was significantly higher in PD patients than in age- and sex-matched healthy controls, and showed a negative correlation to SBR in bilateral putamen and ipsilateral caudate in all PD subjects. Among our three subgroups, only TD patients showed remarkable results. A positive association between NLR and motor severity was observed in TD subgroup. Besides, NLR could negatively predict the SBR in ipsilateral and contralateral putamen and caudate nuclei in tremulous phenotype. Nonetheless, we found no significant association between NLR and other clinical and imaging findings in PIGD and indeterminate subgroups, supporting the presence of distinct underlying pathologic mechanisms between tremor and non-tremor predominant PD at early stages of the disease.

## Introduction

Progressive dopaminergic demise in the nigrostriatal system has long been considered the pathogenic mainstay of motor features in Parkinson’s disease (PD). Lots of ergot mechanisms have been enumerated to be responsible for the neural loss in PD, of which inflammation, both central and peripheral, has been explained in the initiation and progression of PD.

Inflammatory processes, which once were believed to be just the afterclap of neural death, are now considered as an etiological factor, which are also presented in early stages of PD. Several PD susceptibility genes are documented to be involved in the functions of immunity components like microglia (1–3). The result from polymorphism studies determined several pro-inflammatory genes such as IL-1β, TNF-α, HLA-DBQ1, HLA-DRA, and HLA-DRB1 to be associated with an increased susceptibility to PD (4, 5). It is also proved that nonsteroidal anti-inflammatory drugs can in the long term diminish the risk of PD (6). In essence, the authors exhibited that dopaminergic neurons are selectively susceptible to oxidative stress and inflammation (7, 8). Freshly, a neuroimaging study using positron emission tomography detected activated microglia in the SN and putamen of 1-year duration PD patients (9), implying that there is an active inflammatory condition in the initial phases of PD. Furthermore, the authors suggested that inflammatory circumstances, both central and peripheral, are present in the prodromal PD (10, 11). Altogether, these immense lines of evidence highly signify the importance of inflammation in the pathophysiology of PD.

A robust body of evidence has espoused the involvement of peripheral inflammatory conditions in the pathophysiology of PD. In this context, markers of peripheral inflammatory processes including, but not limited to, TNF-α and its receptors and IL-1β have been exhibited to be elevated in individuals with PD in the peripheral blood (12–16), cerebrospinal fluid, and postmortem brain tissues (12, 17–19). Related data showed that IL-6 is associated with an increased risk of the development of PD (20). Besides the peripheral immune markers, peripheral immune cells undergo some quantitative alterations in PD patients. The most prominent of these changes is the lower total number of lymphocytes in individuals with PD compared to controls mainly due to a decrement in the percentage of CD3^+^ and CD19^+^ lymphocytes (21–24). Furthermore, decreased counts of T helper cells and increased/unchanged counts of cytotoxic T cells have been documented in PD patients (25).

Owing to the presence of blood–brain barrier (BBB), central nervous system is an immune-privileged organ. However, BBB dysfunction has been postulated to play a key role in the pathogenesis of neurodegenerative diseases like PD (26, 27). It has been shown that endothelium residing in substantia nigra pars compacta (SNpc) of patients with PD undergoes radical pathologic morphological changes (28, 29), and CD4^+^ and CD8^+^ T-lymphocytes have been reported to enter and invade the SNpc of idiopathic PD patients (30). Using a mouse model involving the injection of vascular endothelial growth factor (a detrimental protein to BBB) into the mice SNpc, researchers demonstrated a significant association between BBB dysfunction and dopaminergic cell death (31, 32). Furthermore, inflammatory cytokines can induce the activation of microglia and therefore the dopaminergic cell death. In this context, one of the valid indicators for peripheral inflammatory processes is the neutrophil to lymphocyte ratio (NLR) (33), representing the active inflammation (neutrophils) merged with immunological regulatory processes (lymphocytes).

Despite lots of evidence on the role of inflammation in dopaminergic neural loss, no direct assessment has been performed in this regard. This work was designed to investigate peripheral inflammation in terms of NLR concerning the loss of dopaminergic uptake in caudate and putamen on Dopamine Transporter Single Photon Emission Computed Tomography (SPECT) (DaTscan) images, which signifies the dopaminergic activity, among treatment-naïve early PD patients with different motor phenotypes and severities.

## Materials and Methods

### Participants

Parkinson’s disease patients and healthy control (HC) subjects enrolled in this study were recruited from the Parkinson Progression Markers Initiative (PPMI, http://www.ppmi-info.org/). The study was approved by the institutional review board of all participating sites. Written informed consent was obtained from all participants before study enrollment. The study was performed in accordance with relevant guidelines and regulations. Only baseline visit data are analyzed in this research. PD status was confirmed by Movement Disorder Society-Unified Parkinson’s Disease Rating Scale (MDS-UPDRS), and dopamine transporter deficit was observed on DAT scans. All PD patients at baseline were drug-naïve, non-demented, at Hoehn and Yahr (H&Y) staging I or II, and were confirmed negative for any medical or psychiatric disorders apart from PD.

### Clinical Assessment and Motor Classification

Motor and non-motor symptoms were evaluated among subjects with clinical tests at baseline visits at each participating site. The most common PD rating scales, i.e., UPDRS, H&Y staging, and the Schwab and England rating of activities of daily living (Modified Schwab & England ADL) were assessed (34). Tremor score and postural instability and gait difficulty (PIGD) score were identified for PD subjects with MDS-UPDRS. Then, PD subjects were classified into three groups of tremor dominant (TD), PIGD, and indeterminate based on the ratio of tremor score/PIGD score. If ratio > = 1.15 or PIGD = 0 and tremor >0, then the subject was TD. If ratio < = 0.9, the subject was classified as PIGD. If ratio >0.9 and <1.15, or tremor score and PIGD score = 0, the subject was tagged as indeterminate (35). Non-motor symptoms were investigated by the Montreal Cognitive Assessment (MoCA) for mild cognitive impairment, 15-item geriatric depression scale for depressive symptoms, The University of Pennsylvania Smell Identification Test for olfaction function, and independent validation of the scales for outcomes in Parkinson’s disease-autonomic (SCOPA-AUT) for autonomic dysfunction.

### Blood Sample Collection

As it is pronounced in the PPMI biologics manual (http://www.ppmi-info.org/), baseline blood sample collection was completed at each study site. PAXgene blood RNA tubes were utilized for the collection of blood samples following the study protocol. The number of neutrophils and lymphocytes was calculated by an autoanalyzer device on participants’ whole blood samples, right after the collection. NLR was simply calculated by dividing neutrophil to lymphocyte count.

### Dopaminergic Imaging

As a preventive measure, all females with the potential of pregnancy were confirmed negative for urine pregnancy test prior to receiving the ^123^Ioflupane SPECT injection. None of the subjects had received any of the drugs that would interfere with DAT SPECT imaging within 6 months of screening (full PPMI study protocol is provided at http://www.ppmi-info.org/study-design/research-documents-and-sops/). DAT imaging at baseline visit from all participating sites was centrally reconstructed and was attenuation corrected, followed by spatial normalization for consistent orientation by experienced nuclear medicine experts. Standard volume of interest template was applied on caudate, putamen, and occipital regions. Striatal binding ratios (SBRs) for left and right putamen and caudate were finally calculated, using the formula: (SBR) = (striatal region)/(occipital) −1, where occipital lobe DAT count is the reference (36). Regarding motor dominancy, SBRs of ipsilateral and contralateral putamen and caudate were compared between PD and HC and within PD motor subtypes. The calculated average of SBRs for the left and the right nuclei is presented for HCs or symmetrical motor involvement in PD individuals.

### Statistical Analysis

The statistical analysis was performed using SPSS version 22 (BM Corp., Armonk, NY, USA). Pearson’s chi-square was used to assess nominal variables across groups. Mann–Whitney *U*-test was used to assess differences between HCs and PD patients, and Kruskal–Wallis test was used for multiple comparisons in three groups of PD motor subtypes. Spearman’s rank-order correlation was used to test the association between NLR and other characteristics in patients with PD. Finally, *P*-values less than 0.05 were considered to be statistically significant.

## Results

### Between Group Comparisons

Neutrophil to lymphocyte ratio and DAT scan data were provided for 388 *de novo* PD patients and 148 HC on baseline visits. Baseline clinical characteristics and detailed demographic data of PD and HC subjects are demonstrated in Table 1. Healthy participants were age- and sex-matched to PD patients, while scored higher on all motor and non-motor tests. As expected, PD patients had significantly lower SBRs in striatal nuclei. NLR was significantly higher in PD cohort.

**Table 1:** Demographic information and comparison of clinical outcomes between HCs and patients with PD.

Characteristic	HCs (*n* = 148)	PD (*n* = 388)	*P*-value
Age, mean (SD) (95% CI), years	61.1 (11.3) (59.3–62.9)	61.7 (9.6) (60.7–62.6)	0.775^a^
Female/male, No. (%male)	49/99 (66.9)	134/254 (65.4)	0.755^b^
Left-handed/right-handed, No. (%right-handed)^c^	22/119 (80.4)	33/346 (89.2)	0.029^b^
Education, mean (SD) (95% CI), years	16.1 (3.0) (15.7–16.6)	15.5 (3.0) (15.2–15.8)	0.034^a^
Disease duration, median (range), m	–	4.0 (0–36)	–
NLR, mean (SD) (95% CI)	2.2 (0.8) (2.1–2.3)	2.5 (0.9) (2.4–2.6)	<0.001^a^
SBR score in caudate nucleus, mean (SD) (95% CI)	3.0 (0.6) (2.9–3.1)	contralateral^d^: 1.8 (0.6) (1.8–1.9)ipsilateral: 2.2 (0.6) (2.1–2.2)	<0.001^a^
SBR score in putamen nucleus mean (SD) (95% CI)	2.1 (0.5) (2.0–2.2)	contralateral: 0.7 (0.3) (0.6–0.7)ipsilateral: 1.0 (0.4) (0.9–1.0)	<0.001^a^
Hoehn and Yahr stage, mean (SD)	–	1.6 (0.5)	–
MDS-UPDRS part I score, mean (SD)	3.0 (2.7)	5.6 (4.0)	<0.001^a^
MDS-UPDRS part II score, mean (SD)	0.4 (1.0)	6.0 (4.2)	<0.001^a^
MDS-UPDRS part III score, mean (SD)	1.2 (2.1)	20.4 (8.8)	<0.001^a^
Modified Schwab & England ADL, mean (SD)	–	93.2 (6.0)	–
MoCA score, mean (SD)	28.2 (1.1)	27.1 (2.3)	<0.001^a^
GDS score, mean (SD)	1.1 (1.5)	2.3 (2.4)	<0.001^a^
UPSIT score, mean (SD)	34.4 (4.5)	22.3 (8.2)	<0.001^a^
SCOPA-AUT score, mean (SD)	5.7 (3.5)	9.4 (6.0)	<0.001^a^

*HCs, healthy controls; PD, Parkinson disease; NLR, neutrophil to lymphocyte ratio; SBR, striatal binding ratios; MDS-UPDRS, Movement Disorder Society-sponsored revision of the Unified Parkinson’s Disease Rating Scale; MoCA, Montreal cognitive assessment; GDS, Geriatric depression scale; Modified Schwab & England ADL, overall activities of daily living; UPSIT, University of Pennsylvania Smell Identification Test; SCOPA-AUT, Scales for Outcomes in Parkinson’s disease*.

*^a^Based on Mann–Whitney U-test*.

*^b^Based on χ2-test*.

*^c^Others were mixed-handed*.

*^d^Contralateral and ipsilateral striatal nuclei are against the side of motor dominancy. P-value for bilateral nuclei is <0.001*.

As shown in Table 2, there was no difference between age, gender, UPDRS-III as well as NLR and SBRs in both ipsilateral and contralateral striatal nuclei among PD motor subtypes.

**Table 2:** Demographic data and comparison of certain characteristics between patients with Parkinson’s disease (PD) based on tremor/postural instability and gait difficulty (PIGD) score ratio.

Characteristic		PIGD^a^ (*n* = 73)	Indeterminate (*n* = 27)	Tremor dominant (*n* = 288)	*P*-value
Age, mean (SD) (95% CI), years		60.8 (9.5) (58.6–63.0)	62.8 (10.0) (58.8–66.8)	61.8 (9.7) (60.7–62.9)	0.744^b^
Female/male, No. (%male)		29/44 (60.0)	6/21 (77.7)	99/189 (65.6)	0.261^c^
MDS-UPDRS part III score, mean (SD) (95% CI)		19.2 (8.7) (17.1–21.2)	18.1 (7.7) (15.1–21.2)	20.4 (9.0) (19.9–22.0)	0.195^b^
NLR, mean (SD) (95% CI)		2.5 (0.8) (2.3–2.6)	2.6 (1.0) (2.2–3.0)	2.5 (1.0) (2.4–2.6)	0.952^b^
SBR in contralateral nuclei, mean (SD) (95% CI)	Caudate	1.8 (0.6) (1.6–1.9)	1.8 (0.5) (1.6–2.0)	1.8 (0.6) (1.8–1.9)	0.631^b^
	Putamen	0.7 (0.3) (0.6–0.8)	0.6 (0.2) (0.5–0.7)	0.7 (0.3) (0.6–0.7)	0.163^b^
SBR in ipsilateral nuclei, mean (SD) (95% CI)	Caudate	2.1 (0.7) (2.0–2.3)	2.1 (0.6) (1.9–2.4)	2.2 (0.6) (2.1–2.2)	0.813^b^
	Putamen	1.0 (0.4) (0.8–1.0)	1.0 (0.4) (0.8–1.2)	1.0 (0.4) (0.9–1.0)	0.713^b^

*NLR, neutrophil to lymphocyte ratio; SBR, striatal binding ratios; MDS-UPDRS, Movement Disorder Society-sponsored revision of the Unified Parkinson’s Disease Rating Scale*.

*^a^The ratio of tremor and PIGD score in MDS-UPDRS. PD patients with a ratio of ≥1.15 were classified as TD, ratio ≤0.90 were classified as PIGD, and 0.90 < ratio < 1.15 were classified as indeterminate*.

*^b^Based on Kruskal–Wallis test*.

*^c^Based on χ2-test*.

### Correlations Between NLR and Other Clinical and Imaging Metrics

Spearman’s rank-order correlation was performed in HCs and three subgroups of PD patients (Table 3). NLR had a strong positive correlation with age and UPDRS-III score and a negative correlation with Modified Schwab & England ADL score only in TD subgroup of PD patients. Moreover, TD patients showed a negative correlation between NLR values and SBR in contralateral putamen (*p*-value of 0.041) and ipsilateral putamen and caudate regarding motor dominancy (*P*-values of <0.001 and 0.005, respectively). No significant correlation was observed between NLR and other features in two other motor phenotypes. Results did not differ after controlling for the effect of age (data not shown).

**Table 3:** The correlation (Spearman) between NLR and other characteristics in HCs and patients with PD based on tremor/PIGD score ratio.

		Age	Disease duration	Tremor score	PIGD score	UPDRS part III score	Modified Schwab & England ADL	UPSIT score	MoCA score	SCOPA-AUT score	SBR score in caudate (contralateral)	SBR score in putamen (contralateral)	SBR score in caudate (ipsilateral)	SBR score in putamen (ipsilateral)
NLR, *P*-value (rho)	HCs (*n* = 148)	0.405 (0.069)	–	–	–	0.388 (−0.072)	–	0.133 (−0.124)	0.704 (−0.031)	0.480 (−0.059)	0.590 (−0.045)	0.702 (−0.032)	–	–
	All PD (*n* = 388)	**0.011 (0.129)**	0.495 (0.035)	0.066 (0.093)	0.662(0.022)	0.058 (0.096)	**0.015 (−0.123)**	0.744 (−0.017)	0.891 (−0.007)	0.185 (0.067)	0.199 (−0.066)	**0.026 (−0.114)**	**0.014 (-0.126)**	**0.002 (−0.158)**
	PIGD^a^ (*n* = 73)	0.991 (0.001)	0.834 (0.025)	0.706 (−0.045)	0.667(0.051)	0.947 (−0.008)	0.695 (0.047)	0.732 (−0.041)	0.232 (0.142)	0.581 (0.066)	0.947 (0.008)	0.780 (−0.033)	0.936 (0.010)	0.942 (0.009)
	Indeterminate (*n* = 27)	0.813 (−0.048)	0.488 (−0.139)	0.867 (0.034)	0.921(0.020)	0.374 (−0.178)	0.058 (−0.370)	0.926 (0.019)	0.524 (0.128)	0.720 (0.072)	0.97 (0.008)	0.287 (−0.213)	0.953 (0.012)	0.659 (−0.089)
	TD (*n* = 288)	**0.003** (0.172)	0.371 (0.053)	0.050 (0.116)	0.603(0.031)	**0.009** (0.155)	**0.015** (−0.144)	0.685 (−0.024)	0.426 (−0.047)	0.214 (0.073)	0.106 (−0.096)	**0.041 (−0.120)**	**0.005 (−0.168)**	**<0.001 (−0.208)**

*HCs, healthy controls; PD, Parkinson’s disease; PIGD, postural instability–gait difficulty; NLR, neutrophil to lymphocyte ratio; UPDRS, Unified Parkinson’s Disease Rating Scale; MoCA, Montreal cognitive assessment; Modified Schwab & England ADL, overall activities of daily living; UPSIT, University of Pennsylvania Smell Identification Test; SCOPA-AUT, Scales for Outcomes in Parkinson’s disease; SBR, striatal binding ratios*.

*^a^The ratio of tremor and PIGD score in MDS-UPDRS. Parkinson’s disease patients with a ratio of ≥1.15 were classified as tremor-dominant (TD), ratio ≤0.90 were classified as PIGD, and 0.90 < ratio < 1.15 were classified as indeterminate*.

*Significant correlations are bolded*.

Eventually, TD patients were tested for possible correlations between NLR and other characteristics, based on their H&Y stage. In 164 TD-PD patients at H&Y stage II, NLR had a positive correlation with age. As TD patients at H&Y stage II were significantly older than TD patients at stage I (Table 4), we carried partial correlation between NLR and SBR in putamen and caudate nuclei, controlling for age. Lower SBRs in bilateral putamen and caudate nuclei were significantly associated with higher NLRs in only the subgroup in stage II (Table 5). No significant correlation was found in 124 TD patients with H&Y stage I.

**Table 4:** Demographic information and comparison of clinical outcomes in patients with Parkinson’s disease (PD) based on H&Y stage.

Characteristic	H&Y stage I (*n* = 124)	H&Y stage II (*n* = 164)	*P*-value
Age, mean (SD), years	59.0 (9.9)	63.9 (8.9)	<0.001^a^
Disease duration, median (range), m	4.0 (1–35)	4.0 (0–36)	0.864^a^
NLR, mean (SD)	2.4 (0.9)	2.7 (1.0)	0.003^a^
SBR score in caudate nucleus (contralateral), mean (SD)	1.9 (0.6)	1.8 (0.5)	0.166^a^
SBR score in putamen nucleus (contralateral), mean (SD)	0.7 (0.3)	0.7 (0.2)	0.032^a^
SBR score in caudate nucleus (ipsilateral), mean (SD)	2.3 (0.6)	2.1 (0.5)	0.004^a^
SBR score in putamen nucleus (ipsilateral), mean (SD)	1.1 (0.4)	0.9 (0.3)	<0.001^a^
Tremor score, mean (SD)	0.5 (0.2)	0.6 (0.3)	0.017^a^
PIGD score, mean (SD)	0.1 (0.1)	0.2 (0.2)	0.056^a^
MDS-UPDRS part III score, mean (SD)	15.1 (5.4)	25.4 (8.5)	<0.001^a^
Modified Schwab & England ADL, mean (SD)	95.2 (5.1)	92.0 (5.8)	<0.001^a^
MoCA score, mean (SD)	27.3 (2.2)	26.8 (2.4)	0.074^a^
GDS score, mean (SD)	2.1 (2.5)	2.4 (2.3)	0.081^a^
UPSIT score, mean (SD)	23.7 (8.1)	21.0 (8.0)	0.006^a^
SCOPA-AUT score, mean (SD)	9.0 (6.4)	10.3 (6.1)	0.051^a^

*H&Y, Hoehn and Yahr stage; NLR, neutrophil to lymphocyte ratio; SBR, striatal binding ratios; PIGD, postural instability–gait difficulty; MDS-UPDRS, Movement Disorder Society-sponsored revision of the Unified Parkinson’s Disease Rating Scale; MoCA, Montreal cognitive assessment; GDS, Geriatric depression scale; Modified Schwab & England ADL, Overall activities of daily living; UPSIT, University of Pennsylvania Smell Identification Test; SCOPA-AUT, Scales for Outcomes in Parkinson’s disease*.

*^a^Based on Mann–Whitney U-test*.

**Table 5:** The correlation (Spearman) between NLR and other characteristics in patients with tremor-dominant (TD) Parkinson’s disease (PD) based on H&Y stage.

											Contralateral^a^ nuclei		Ipsilateral nuclei	
		Age	Disease duration	Tremor score	PIGD score	UPDRS part III score	Modified Schwab & England ADL	UPSIT score	MoCA score	SCOPA-AUT score	SBR score in caudate	SBR score in putamen	SBR score in caudate	SBR score in putamen
NLR, *P*-value (rho)	H&Y stage I (*n* = 124)	0.902 (−0.011)	0.548 (−0.055)	0.563 (0.052)	0.242 (−0.106)	0.486 (0.063)	0.064 (−0.167)	0.263 (0.101)	0.754 (0.028)	0.980 (0.002)	0.738 (−0.030)	0.947 (−0.006)	0.640 (−0.043)	0.541 (−0.056)
	H&Y stage II (*n* = 164)	**0.002** (0.240)	0.088 (0.134)	0.140 (0.116)	0.217 (0.097)	0.278 (0.085)	0.352 (−0.073)	0.292 (−0.083)	0.338 (−0.075)	0.241 (0.092)	**0.040 (−0.162)**	**0.017 (−0.188)**	**0.003 (−0.221)**	**0.019 (−0.185)**

*H&Y, Hoehn and Yahr stage; NLR, neutrophil to lymphocyte ratio; UPDRS, Unified Parkinson’s Disease Rating Scale; MoCA, Montreal cognitive assessment; Modified Schwab & England ADL, overall activities of daily living; UPSIT, University of Pennsylvania Smell Identification Test; SCOPA-AUT, Scales for Outcomes in Parkinson’s disease; SBR, striatal binding ratios*.

*^a^Numbers for all SBRs are representative of added partial correlation between NLR and SBR in putamen and caudate nuclei, controlling for age*.

*Significant correlations are bolded*.

## Discussion

In search of possible correlation between peripheral inflammation and striatal DAT availability in a large sample of drug-naïve early PD patients, we found that (1) NLR is significantly higher in PD patients than in age- and sex-matched HCs. (2) Although NLR and striatal SBR did not differ between subtypes of PD in the early stages of the disease, only TD phenotype and more specifically at H&Y stage II confirmed predictive value of NLR to dopaminergic loss in bilateral striatal nuclei. (3) Worse motor severity was associated with higher NLR values in only TD phenotype.

Tremor is a cardinal motor feature of PD that has always exhibited unalike clinical course to other motor phenotypes. In contrast to TD, non-TD subtypes, especially PIGD, predict worse and rapid disease progression with higher rates of non-motor features such as cognitive decline, mood disturbances, REM sleep behavior disorder, and olfaction dysfunction (37–42). A longitudinal study has revealed that TD patients will not develop dementia until irreversibly converted to PIGD phenotype (43). Differed clinical pattern is also reflected on structural and functional assays. Autopsy of TD-PD patients has exposed less severe dopaminergic loss in SN than other PD subtypes (44, 45). This is in concordance with a slower progression of symptoms in this group (37, 46) and is replicated on a recent probabilistic tractography study, which has revealed normal structural connectivity in nigro-pallidal and fronto-striatal circuits in TD patients, which was altered in non-TD (47). Postmortem studies have also demonstrated distinct striatal pathology among different PD motor subtypes. While akinesia and rigidity evolve from cell loss in ventrolateral SNpc which project to the posterior putamen, degeneration in TD is most prominent in dorsomedial SNpc projecting to the caudate and anterior putamen and to the retrorubral field with subsequent projections to the dorsolateral striatum and ventromedial thalamus (48). In support of this model, Eggers et al. showed lesions in caudate and lateral putamen (eagle-wing shape) on DAT scans of TD patients, compared to lesions in dorsal putamen with an egg-shape pattern of dopaminergic loss in akinetic-rigid phenotype (49). This pattern is consistent with our finding of NLR correlation to lower dopamine uptake in both putamen and caudate in TD subgroup, while it was linked to a lower DAT binding in contralateral putamen and not contralateral caudate in all PD patients. In fact, there is consistent report of a higher extent of dopamine depletion in putamen than caudate in PD (50). Moreover, it is proposed that tremor severity may have a different mechanism than other motor features, as in contrast to bradykinesia and rigidity, does not depend on the degree of reduced striatal dopamine uptake (50–53), but rather on the relative contribution of a reduced dopamine in putamen and caudate, indicative of a more severe caudate involvement (51). Therefore, a strong correlation of NLR to SBR in caudate of TD patients, but a weak association with putamen (Table 5), agrees with these sightings. In this study, we did not find any difference between SBR values in ipsilateral or contralateral caudate and putamen nuclei between three examined subtypes with similar motor stage and severity. However, early drug-naïve TD patients of another study have shown higher DAT availability on bilateral putamen but no difference on caudate nuclei compared to akinetic-rigid PD patients with higher UPDRS-III and H&Y (54). This may point to the possibly greater impact of putamen on motor severity in non-TD phenotype. In this study, we did not investigate other neurotransmitters than dopamine which are conferred regarding tremorgenesis, and its severity is shown to be mediated by reductions of serotonin transporter availability in the raphe nuclei more than that of dopamine in putamen (55, 56), or mediated by neurotransmitter imbalance between reduced GABAergic inhibitory influx from putamen confronted to intact dopaminergic input from SN to the internal pallidum (57).

On the other hand, we found surprisingly much more substantial reduction in DAT availability in ipsilateral putamen and caudate and a weak reduction in contralateral nuclei in TD patients at H&Y stage II compared to TD at stage I. This is also true for the strong correlation of NLR to ipsilateral caudate (*p*-value = 0.003) and a weak relationship with contralateral caudate nucleus (*p*-value = 0.04). This is the same for ipsilateral versus contralateral putamen among all PD patients as well as all individuals tagged in TD subtype. Motor symptoms in PD are considered to evolve from dopaminergic depletion in contralateral nigrostriatal system. However, there are several recent reports of motor dominancy ipsilateral to the side of predominant dopaminergic deficit, especially in TD patients (58–60). This is also true for medication-naïve TD patients from PPMI cohort on early stages of the disease (61). Another study on PPMI cohort (56) disclosed that a proportion of TD group at baseline progressed to bilateral limb involvement over 2 years of follow-up, which may be due to the observed ipsilateral striatal dopamine depletion in this study. It is of particular note that TD patients showed negative association between NLR and ipsilateral caudate, and while splitting them based on H&Y stage, contralateral caudate emerged to have such an association only in the group at stage II. More studies are needed to further elucidate the contribution of ipsilateral basal ganglia motor circuits to the tremorgenesis.

Outside the nigrostriatal tract, imaging studies have imposed the increased functional and metabolic activity of cerebellothalamocortical pathway in emerging parkinsonian tremor, which largely drives by depleted dopaminergic input from basal ganglia (62–64). In a recent diffusion tensor imaging study, Wen et al. have revealed that drug-naïve early TD patients recruited from PPMI project have a greater white matter integrity and a lesser neural degeneration, while they observed a widespread white matter dehiscence in PIGD group with similar motor stage and severity. The authors have discussed this finding as a neural compensation for nigrostriatal dopaminergic loss leading to more benign disease course in TD subtype. In addition, white matter alterations were also more significantly related to the severity of symptoms in PIGD than that of TD (65).

Overall, the different pattern of clinical course and dopaminergic loss along with the consistent findings of the involvement of distinct neural circuits outside the nigrostriatal region strongly suggest the different underlying pathophysiology of PD tremor from that of other motor features (66). This study is the first that has captured the different pathogenesis of the dopaminergic neuronal loss in terms of higher NLR interrelated to lower striatal dopamine in TD but not in non-TD PD. NLR is an easy accessible marker of peripheral inflammation, and is identified as a predictor of worse outcome in multiple chronic clinical and neurological disorders, which are more prevalent in geriatric population. This marker also positively correlates to age in normal population (67). Only two previous studies have investigated the association of NLR in idiopathic PD with opposite results. Akil et al. have found higher NLR in 51 PD (NLR = 3.1 ± 1.3) compared to 50 HC (NLR = 2.1 ± 10.3) (68), while Uçar et al. did not find any difference comparing 46 PD (NLR = 2.66 ± 1.05) and 60 HC (NLR = 2.46 ± 1.04) (69). The later study also did not find any difference between TD and akinetic-rigid subtypes. These studies have assessed relatively small sample sizes of PD patients already on dopaminergic replacement therapy, which is shown to recover changes on T-lymphocytes (70). In this study, we surveyed a larger sample of 388 drug-naïve PD patients who showed higher levels of NLR (2.5 ± 0.9) compared to 148 age-matched HCs (NLR = 2.2 ± 0.8), although there was no such difference within PD subtypes. NLR is also shown to be attributed to a lower connectivity in several white matter tracts implicated in early or prodromal PD pathology (71).

Several studies have manifested that PD progression does not follow a linear model, and a decline in striatal uptake observed on DAT scans is more rapid in early stages of the disease followed by a much more slower progression in advanced stages (72–74). Therefore, it seems that early stages of the disease without the interference of dopaminergic replacement therapy are the best point during the disease course to evaluate the contribution of inflammation to dopaminergic loss. A growing body of evidence suggests that presynaptic terminals in dorsal striatum undergo degeneration even prior to the SN, and axonal degeneration should be the primary target for neuroprotective therapies (75). This is supported by *in vivo* DAT SPECT (76) and confirms that lower SBRs in putamen and caudate nuclei truly reflect the nigrostriatal pathology in early stages of PD. However, study on early stages imposes some limitations that should be considered when interpreting our results. First, motor classification tends to change during the course of the disease, and our subtype classification was only based on screening visits. Second, tremor predominant PD patients recognize the symptoms and seek medical advice earlier and owing to the more benign course they more readily cooperate with PPMI project that includes patients not receiving any parkinsonian treatment for 6 months. Therefore, a higher number of cases tagged in TD subgroup and relatively fewer cases in PIGD and indeterminate in PPMI cohort may have contributed to the false-negative results in non-tremor predominant groups. More studies are clearly needed to investigate the generatability of these solid findings.

## Conclusion

In this research, we investigated the association between immune and nervous system. PD patients were divided into three groups based on tremor/PIGD score, being TD, PIGD, and indeterminate subgroups. We demonstrated that NLR can negatively predict SBR in bilateral putamen and caudate nuclei of TD group and more specifically at H&Y stage II. Not observing such association in non-tremor predominant PD patients points to the different pathophysiology mechanisms between tremor and other motor symptoms in PD. Making a bridge between inflammation and neurodegeneration, it is suggested that peripheral inflammation can potentially contribute to the initiation and progression of PD, particularly the process of dopaminergic depletion in striatal regions. However, this relation between peripheral inflammation and Parkinson progression is not conclusive. Much more studies are required to further investigate this subject.

## Availability of Data and Materials

All relevant data are available in the Parkinson’s Progression Markers initiative (PPMI) database (http://www.ppmi-info.org/data).

## Ethics Statement

All procedures performed here, including human participants, were in accordance with the ethical standards of the institutional research committee and with the 1964 Helsinki declaration and its later amendments or comparable ethical standards. Informed consent was obtained from all individual participants included in the study.

## Author Contributions

HSM, FGS, MMZ, and MA contributed to the conception and design of the study. AA-G, and MA contributed to data collection and analysis. HSM, FGS, MMZ, and MA contributed to writing the manuscript. FGS contributed to revising of the final draft.

## Conflict of Interest Statement

The authors declare that the research was conducted in the absence of any commercial or financial relationships that could be construed as a potential conflict of interest.
